# 2072. Of Mice and Men: HIV-induced CD38 expression on CD8+ T lymphocytes contributes to mitochondrial dysfunction and chronic inflammation despite antiretroviral therapy

**DOI:** 10.1093/ofid/ofac492.1694

**Published:** 2022-12-15

**Authors:** Poonam Mathur, Shyam Kottilil, Alonso Heredia, Alip Ghosh, Ben Atkinson

**Affiliations:** University of Maryland, Baltimore, MD; Institute for Human Virology (IHV), University of Maryland School of Medicine, Baltimore, Maryland; Institute of Human Virology, University of Maryland School of Medicine, Baltimore, Maryland; Institute of Human Virology, University of Maryland School of Medicine, Baltimore, Maryland; Institute of Human Virology, University of Maryland School of Medicine, Baltimore, Maryland

## Abstract

**Background:**

Antiretroviral treatment (ART) has increased the life expectancy of people with HIV (PWH), however, comorbidities are higher in PWH than HIV-negative individuals. This is partly due to accelerated cellular aging, a consequence of CD38 expression, mitochondrial dysfunction, and inflammation. However, the role of CD38-expressing T cells on accelerated cellular aging in PWH is not completely understood.

**Methods:**

The proportion of cytotoxic CD38+ CD8+ T lymphocytes from peripheral blood mononuclear cells (PBMCs) of PWH and HIV negative-individuals was measured. PBMCs from PWH on ART were cultured with or without gag-specific peptide stimulation and cytokine production was detected by flow cytometry. Mitochondrial function from T cells of PWH on ART and healthy donors was compared. Last, we generated a humanized (NSG) mouse model and infected mice with HIV (Bal strain) to determine the proportion of CD38+ T cells and cytokine production.

**Results:**

Mean CD38 expression on CD8+ T cells was significantly different between PWH on ART and HIV-negative individuals (Figure 1). In PWH, gag-specific peptide stimulation of CD8+ T cells significantly increased CD38 expression (Figure 2A) and augmented secretion of IFNγ+ and TNFα+ (Figure 2B). There was a higher expression of CD38 among CD8+ T cells that produced IFNγ and TNFα (Figures 2C). T cells in PWH have altered mitochondrial function (Figures 2D-2F). Finally, HIV-infected NSG mice had T cell changes consistent with HIV infection (Figure 3A), a higher proportion of CD38+ T cells with an activated phenotype (Figures 3B and 3C), and significantly higher levels of inflammatory cytokines (Figure 3D).

**Figure 1.**
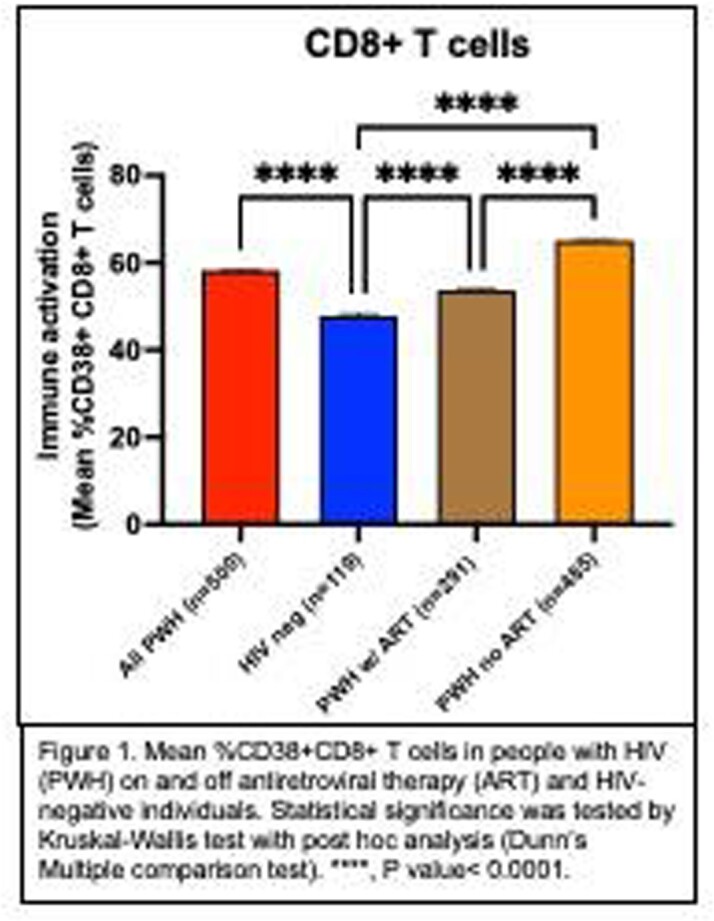
CD38 expression in PWH and HIV-negative individuals.

**Figure 2.**
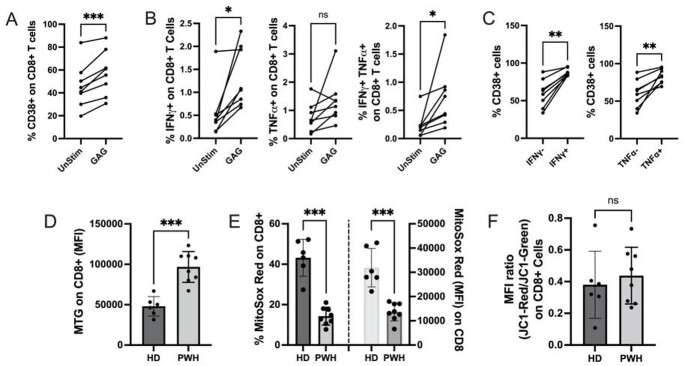
Cytokine production in response to gag stimulation from CD38+CD8+ T cells and mitochondrial function in T cells of PWH.

Figure 2. (A) Frequency of CD38+ cells in gag-peptide stimulated CD8+ T cells and unstimulated cells. (B) Frequency of IFNγ and TNFα producing CD8+ T cells in gag-stimulated and unstimulated cells. Proportion of CD38+ cells among (C) IFNγ-/IFNγ+ and TNFα-/TNFα+ gag-peptide stimulated CD8+ T cells. Statistical significance was tested by paired t test. (D) Mitochondrial mass was determined by measuring the mean fluorescence intensity (MFI) of mitochondria-specific dye mitotracker green (MTG) among CD8+ T lymphocytes. (D) Frequency of mitochondrial superoxide positive lymphocytes and level of mitochondrial superoxide (MFI) were determined by staining the CD8+ T lymphocytes with mitosox red dye. (F) Mitochondrial membrane depolarization was determined by the red to green ratio of MFIs of mitochondrial membrane potential-dependent dye JC-1 among the CD8+ T lymphocytes. Statistical significance was tested by Mann-Whitney test. *, P value< 0.05; **, P value< 0.01; ***, P value< 0.001; ns – not significant. PWH=People with HIV. HD= Healthy Donors.

**Figure 3.**
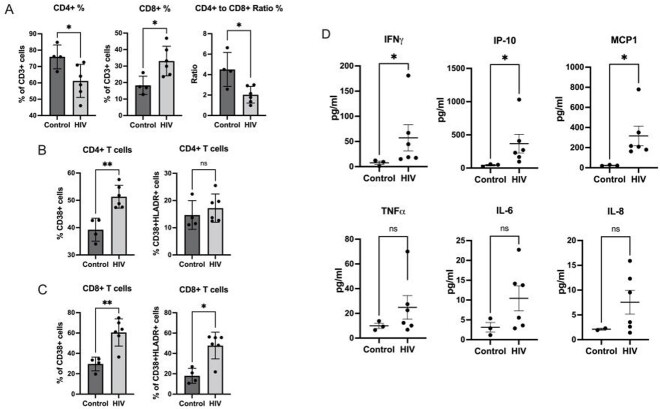
Lymphocyte changes, CD38 expression, and cytokine production in HIV-infected NSG mice.

**Conclusion:**

ART decreases CD38 expression on CD8+ T cells, but not to the levels of HIV-negative individuals. In vitro, T cells of PWH have altered mitochondrial function, and HIV-specific stimulation augments CD38 expression on CD8+ T cells and contributes to a proinflammatory response. These findings translate to a humanized mouse model, where HIV infection upregulates CD38 expression and cytokine production. Together, these data suggest that CD38 is a potential therapeutic target for mitigating the mitochondrial dysfunction and chronic inflammation that drive cellular aging and comorbidities in PWH.

**Disclosures:**

**Poonam Mathur, DO, MPH**, American Academy of HIV Medicine: Honoraria|Med Learning Group: Speaker/presenter|NACCME: Advisor/Consultant|NKGP Pharma: Advisor/Consultant **Shyam Kottilil, MD, PhD**, Arbutus Pharmaceuticals: Grant/Research Support|Gilead: Grant/Research Support|Merck: Grant/Research Support|Regeneron Pharmaceuticals: Advisor/Consultant|Silverback Therapeutics: Advisor/Consultant|The Liver Company: Advisor/Consultant|Yufan Biotechnologies: Advisor/Consultant.

**Figure 1.** CD38 expression in PWH and HIV-negative individuals.

Mean %CD38+CD8+ T cells in people with HIV (PWH) on and off antiretroviral therapy (ART) and HIV-negative individuals. Statistical significance was tested by Kruskal-Wallis test with post hoc analysis (Dunn’s Multiple comparison test). ****, P value< 0.0001.

**Figure 3.** Lymphocyte changes, CD38 expression, and cytokine production in HIV-infected NSG mice.

(A) Frequency of CD4+ and CD8+ T lymphocytes and comparison of ratio of the frequencies of CD4+ to CD8+ T cells between control and HIV infected humanized mice. Statistical significance determined by Mann-Whitney test. (B) Frequency of CD38+ and CD38+HLADR CD4+ (A) and CD8+ (B) T cells in HIV- infected humanized mice compared to control. Statistical significance determined by Mann-Whitney test. (D)Cytokine measurements of HIV-infected humanized mice and controls. Statistical significance was tested by independent t test. *, P value< 0.05; **, P value< 0.01; ns – not significant. HLADR= Human Leukocyte Antigen – DR isotype, IFN=Interferon, IP=Inducible Protein, MCP=Monocyte Chemoattractant Protein, TNF=Tumor Necrosis Factor, IL=Interleukin.

